# Differential effects of lipid biosynthesis inhibitors on Zika and Semliki Forest viruses

**DOI:** 10.1016/j.tvjl.2017.10.009

**Published:** 2017-12

**Authors:** Jamie Royle, Claire L. Donald, Andres Merits, Alain Kohl, Margus Varjak

**Affiliations:** a MRC -University of Glasgow Centre for Virus Research, Glasgow G61 1QH, Scotland, UK; b Institute of Technology, University of Tartu, Nooruse 1, 50411 Tartu, Estonia

**Keywords:** Zika virus, Semliki Forest virus, Lipid biosynthesis, Antiviral agent

## Abstract

The recent outbreak of infection with Zika virus (ZIKV; *Flaviviridae*) has attracted attention to this previously neglected mosquito-borne pathogen and the need for efficient therapies. Since flavivirus replication is generally known to be dependent on fatty acid biosynthesis, two inhibitors of this pathway, 5-(tetradecyloxyl)-2-furoic acid (TOFA) and cerulenin, were tested for their potentiality to inhibit virus replication. At concentrations previously shown to inhibit the replication of other flaviviruses, neither drug had a significant antiviral affect against ZIKV, but reduced the replication of the non-related mosquito-borne Semliki Forest virus (*Togaviridae*).

Arboviruses are a diverse group of viruses that are transmitted by arthropod vectors, including mosquitoes, ticks and midges and belong to four main viral families (*Togaviridae*, *Flaviviridae*, *Bunyaviridae* and *Reoviridae*). In the *Flaviviridae* (genus *Flavivirus*), important and/or representative viruses include Zika virus (ZIKV) and dengue virus (DENV), whilst the genus *Alphavirus* in the *Togaviridae* family contains chikungunya virus (CHIKV) and Semliki Forest virus (SFV). Arbovirus infections can cause severe disease in a range of human and animal hosts, resulting in a significant social and economic impact in many areas of the world (Weaver and Reisen, 2010).

The recent outbreak of infection with Zika virus (ZIKV, *Flaviviridae*) in human beings has brought increased attention to previously this neglected arboviruses. ZIKV is thought to be mainly spread by *Aedes aegypti* mosquitoes in the South American outbreak (Ferreira-de-Brito et al., 2016). Symptoms of infection in human beings are generally mild, consisting of fever and joint pain. However, infection during pregnancy can cause microcephaly and other manifestations that are grouped as Zika congenital syndrome, while infection in adults has been linked to Guillain-Barré syndrome (Russo et al., 2017).

The replication of several flaviviruses, including DENV and alphaviruses (CHIKV), is dependent on continuous synthesis of fatty acids; the use of chemicals to inhibit their production has been shown to decrease virus production (Heaton et al., 2010, Karlas et al., 2016). These pathways are an attractive target for antiviral agents, as has been postulated for flaviviruses, including ZIKV (Martin-Acebes et al., 2016). Fatty acid synthesis in the cytoplasm of eukaryotic cells requires three different enzymes that act in an orchestrated manner: ATP citrate lyase (ACLY), acetyl-CoA carboxylase (ACC) and fatty acid synthase (FASN). Here we aimed to assess if ZIKV replication could be restricted through the treatment of infected cells with drugs that inhibit fatty acid biosynthesis. To this end we used 5-(tetradecyloxyl)-2-furoic acid (TOFA; Sigma–Aldrich) and cerulenin (Sigma–Aldrich), which inhibit ACC and FASN, respectively.

Firstly, the toxicity of the drugs was tested on adenocarcinomic human alveolar basal epithelial (A549) cells grown in Dulbecco’s modified Eagle’s medium (DMEM) supplemented with 10% foetal bovine serum. Cell viability was assessed by measuring ATP levels using the CellTitre-Glo Luminescent Cell Viability Assay (Promega). At 8 h after the addition of the drug, cells treated with cerulenin exhibited reduced viability. This effect was more prominent after 24 h. However, treatment with TOFA did not decrease cell viability (Fig. 1A, top and lower panels).

**Fig. 1:**
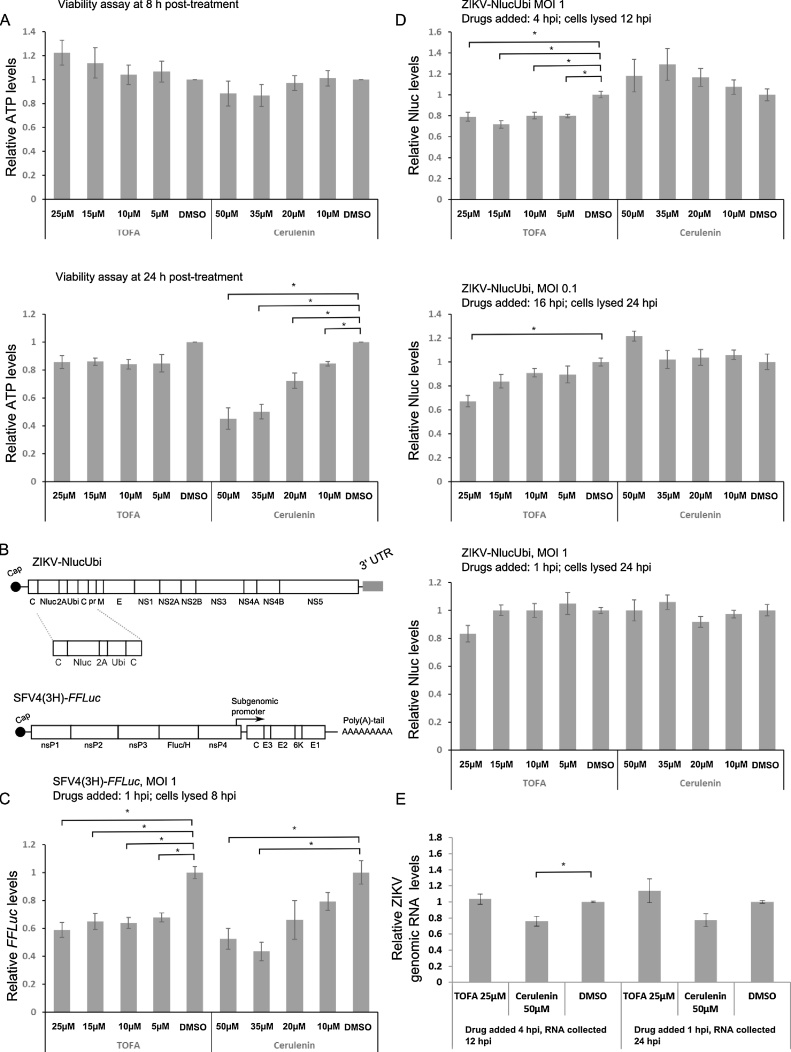
(A) A549 cells were treated with different concentrations of drugs for 8 or 24 h. Dimethyl sulphoxide (DMSO) was used as a negative control and cell viability was measured using a CellTitre-Glo Luminescent Cell Viability Assay kit. The experiment was repeated independently three times. (B) Representation of reporter viruses: (1) Zika virus expressing nanoluciferase (ZIKV-NlucUbi); and (2) Semliki Forest virus expressing firefly luciferase (SFV4(3H)-*FFLuc*). (C) A549 cells were infected with SFV4(3H)-*FFLuc* at a multiplicity of infection (MOI) of 1 and drugs were added at 1 h post-infection (hpi). After 8 hpi, the cells were lysed and firefly luciferase (*FFLuc*) activity was measured. (D) A549 cells were infected with ZIKV-NLucUbi at MOIs of 0.1 or 1 and drugs were added at 1, 4 or 16 h. Cells were lysed after either 12 or 24 hpi and nanoluciferase (Nluc) activity was measured. Experiments in (C) and (D) were conducted independently twice in quadruplicate; the relative mean values with standard errors are depicted. (E) A549 cells were infected with Brazilian ZIKV strain PE243 at a MOI of 1. Drugs were added either 1 or 4 hpi; total RNA was isolated at 12 or 24 hpi, respectively. Genomic RNA levels were analysed by reverse transcriptase quantitative PCR (RT-qPCR) using cellular glyceraldehyde 3-phosphate dehydrogenase (GAPDH) gene as an internal control. The experiment was repeated independently three times and the relative mean values with standard error are depicted. *P < 0.01 in Student’s *t* test.

Secondly, to study the inhibitory effect of these drugs on ZIKV replication, A549 cells were infected with a generic Asian strain-derived ZIKV expressing nanoluciferase (Nluc) followed by foot-and-mouth disease virus 2A autoprotease, ubiquitin sequence (Fig. 1B); these elements are required to liberate Nluc from duplicated capsid protein C. The cells were infected at a multiplicity of infection (MOI) of 0.1 or 1 and the drugs applied at either 4 or 16 h post-infection (hpi), respectively. Due to the toxicity of cerulenin, cells were lysed 8 h post-treatment and Nluc activity was measured using the Nano-Glo kit (Promega). We also compared ZIKV to a recombinant strain of SFV expressing cleavable firefly luciferase (*FFLuc*; designated SFV4(3H)-*FFLuc*) (Fig. 1B). A549 cells were infected at a MOI of 1 and drugs were added 1 hpi.

As expected, both cerulenin and TOFA inhibited SFV; this effect was concentration dependent, since higher concentrations resulted in reduced luciferase amounts (Fig. 1C). Similar effects have been shown previously for CHIKV (Karlas et al., 2016), a close relative of SFV. The results of TOFA treatment on ZIKV showed that, if Nluc levels were measured 12 hpi (Fig. 1D, MOI 1, drugs added at 4 hpi) at the highest drug concentration, only a 20% reduction in Nluc levels was observed, thus indicating a weak decrease in ZIKV replication. This weak effect was also present when cells were lysed at 24 hpi (Fig. 1D, MOI 0.1, drugs added at 16 hpi). However, regardless of the concentration of cerulenin used, there was no decrease in Nluc levels. This suggested there was no effect of this drug on ZIKV replication.

These results were unexpected, since the dependence of flaviviruses on lipid biosynthesis is well documented. We therefore performed a further experiment, whereby A549 cells were infected at a MOI of 1, with drugs added at 1 hpi and Nluc activity measured at 24 hpi. TOFA was again found to have antiviral effects only at the highest concentration, resulting in a weak, non-significant, 20% reduction in luciferase (Fig. 1D). However, despite its observed toxicity, cerulenin did not decrease virus replication.

To confirm these findings, cells were infected with the wild-type Brazilian strain ZIKV PE243 (Donald et al., 2016) at an MOI of 1. Cells were then treated with both drugs either at 1 or 4 hpi and total RNA was collected at 12 or 24 hpi. Using reverse transcriptase (RT) quantitative PCR (RT-qPCR), we found that neither cerulenin nor TOFA decreased virus genomic RNA levels significantly (except for a weak reduction at 12 hpi with cerulenin), thereby suggesting that neither drug affects virus replication (Fig. 1E). The resistance of ZIKV in A549 cells under the conditions tested here was unexpected, since dependence on ACC and FASN has been shown for different flaviviruses including DENV and hepatitis C virus (HCV) (Yang et al., 2008, Heaton et al., 2010). However, both DENV and HCV infections are known to increase production of fatty acids and higher levels of FASN expression.

It is therefore possible that heightened production of FASN could occur in ZIKV infected cells, which may result in relative insensitivity to drugs. Both drugs had the expected antiviral effects against SFV infection and targeting fatty acids synthesis may be one way to treat alphavirus infections. However, a more detailed analysis of the metabolic activities in cells associated with ZIKV is required.

## Conflict of interest statement

None of the authors of this paper has a financial or personal relationship with other people or organisations that could inappropriately influence or bias the content of the paper.
